# Differences in IgE mediated basophil degranulation induced by proteic fractions from whole flea body extract in patients with papular urticaria by flea bite and healthy controls

**DOI:** 10.1186/1939-4551-6-10

**Published:** 2013-05-16

**Authors:** Omar Dominguez-Amorocho, Silvia Duarte, Elizabeth Garcia, Evelyne Halpert, Adriana Cuellar, Adriana Rodriguez

**Affiliations:** 1 Facultad de Medicina, Universidad Militar Nueva Granada, Bogotá, Colombia; 2 Departamento de Microbiología, Facultad de Ciencias, Pontificia Universidad Javeriana, Bogotá, Colombia; 3 Sección de Alergia y Dermatología Pediátrica, Fundación Santa Fe de Bogotá, Carrera 9 Nº 117-20, oficina 205, Bogotá, DC, Colombia; 4 Grupo de Inmunobiología y Biología celular, Facultad de Ciencias, Pontificia Universidad Javeriana, Bogotá, Colombia; 5 Centro de Investigaciones Odontológicas, Facultad de Odontología, Pontificia Universidad Javeriana, Bogotá, Colombia

**Keywords:** Basophil degranulation, CD63, Flea allergy, Papular urticaria, Basophil degranulation test

## Abstract

**Background:**

Papular urticaria by flea bite (PUFB) is a chronic inflammatory disease in children. The aim of this study was to assess the functional activity of IgE to protein fractions from flea body extract, through basophil degranulation in PUFB patients and controls.

**Methods:**

Basophil degranulation, measured by overexpression of CD63 surface molecules, was evaluated by flow cytometry in samples from patients and controls. Cell stimulation was performed with three fractions with different molecular weight from flea body extract using a Basotest® modified protocol. Mann–Whitney *U*-test was used for comparisons.

**Results:**

Specific IgE from PUFB patients and healthy controls induced basophil degranulation to flea body extract with no significant differences between them (16.2 ± 3.1% vs 13.6 ± 2.8% p = 0.77). However, when flea extract was analyzed in fractions with proteins ranging different molecular weights, significant differences were observed on the response from patients compared with controls to <50 kD (14.9 ± 5.1% vs 9.7 ± 2.1% p = 0.0058) and 50–100 kD proteic fractions (8.3 ± 3.2% vs 2.8 ± 1.6% p = 0.0021).

**Conclusion:**

In this study, was established that the differential response by IgE, in PUFB, depends from the molecular weight of the antigens contained in the flea extract. These antigens may be related to 30–35 kD proteins previously described as major allergens.

## Introduction

Papular urticaria by flea bite (PUFB) is a chronic inflammatory disease clinically observed during the first years of life, as a cutaneous hypersensitivity reaction [1]. In Bogotá, Colombia, children with papular urticaria due to flea bites, are part of the dermatologic practice all year round. With its wet and temperate climate, Bogota is an environment where fleas thrive, and their bites afflict to the children. PUFB prevalence in child population between one to six years old in Bogota is 20.3%. The major risk factor for this disease is the presence of fleas at home. With this high prevalence, this disease is important in terms of public health. (Halpert E, Borrero E, Chaparro P, et al.: Prevalencia de la urticaria papular por picadura de pulga y factores asociados en niños de 1–6 años en la ciudad de Bogotá. Informe final Colciencias. Código 622145921508. Article in preparation).

Previous studies showed a pattern of cellular infiltration in the lesions consisting primarily of eosinophils and CD4^+^ T lymphocytes [2] and also it has been observed a predominant Th2 response to a polyclonal stimulus [3]. In addition, functional differences in dendritic cells derived from monocytes in patients compared to those originated from healthy controls, have been also described [4].

The total flea extract contains a large number of proteins with a wide range of molecular weight whose are recognized by IgE from PUFB patients and healthy controls. Proteins with molecular weights ranging 30 and 35 kDa were recognized by nearly 60% of patients. In addition, most of the recognized proteins by patients and controls, were those under 90 kDa [5]. Despite the advance in knowledge of the disease immuno-pathogenesis, at this moment diagnostic and treatment tools are not available for patient intervention.

Beside the complexity of the allergic reaction, the role of the allergen-induced specific IgE, and its binding properties with receptors from cells like basophils, is completely clear. This leucocyte population is present in a very low frequency in peripheral blood but plays an important role as effector cells in the allergic response. Basophils are characterized by a high expression of the tetrameric form of the high affinity receptor for IgE, and they can be activated by allergens in an IgE dependent pathway to release inflammatory mediators as histamine, leukotrienes and Th2 cytokines (IL-4 and IL-13) [6]. The main feature of these cell population, the histamine release in response to the antigen, it has facilitated the studies of the immune response to allergens compared with the study of the tissue mast cells [7].

The purpose of this study was to explore the capacity of allergens with different molecular weights to induce IgE mediated basophil degranulation. Bearing in mind the recognition pattern, proteins from flea body extract were fractioned, according their molecular weight, in <50 kDa, 50 – 100 kDa and >100 kDa.

## Methods

### Study population

It was done an observational analytical, cross-sectional study, that included ten children with ages ranging from two to ten years old (mean age 6.1 ± 3.2) with a clinical diagnosis of papular urticaria induced by the flea bite (PUFB), living in Bogota during the last year. Study participants were recruited from the Pediatric Dermatology and Allergy service in the Fundación Santa Fe de Bogotá, in Bogotá, Colombia. As a control group, we included 10 children with similar age range (mean age 5.9 ± 2.9) whom attended the same institution for minor surgical procedures related to non-inflammatory pathologies. None of the controls had a history of PUFB. An informed consent form was signed by parents or guardians of the children. The study was approved by the Institutional Review Boards of the Fundación Santa Fe de Bogotá, in Bogotá, Colombia.

### Disease diagnosis

The diagnosis of PUFB was carried out by clinical findings, according the morphologic parameters that characterize the disease. Briefly, papular urticaria from flea bites, usually presents as grouped, pruritic lesions, ranging through an oval urticarial flare, small solid papules, vesicles, bullae, and crusted impetigo lesions to residual macules and small pigmented areas. Individual papules may surround a wheal and often have a central punctum or may have small central vesicles in them. The eruption of papular urticaria often shows lesions of varied type at the same time. The individual spots persist and go through a process of resolution which takes several days until they are excoriated or crusted. They are located where clothing fits snugly, such as the sock line and waist band. Exposed areas of the extremities are also commonly affected [1].

### Flea antigen

Aqueous extracts (20% weight/volume) of complete flea (*Ctenocephalides felis*) (Greer Labs, Lenoir, NC, USA) were prepared. The fleas were stored at -70°C until processing time; they were macerated in PBS and shook constantly for 1 hour. The macerated was centrifuged at 15.000 rpm for 15 minutes at 4°C and then it was passed through a 0.22 μm filter to ensure sterility. Protein concentration was determined by Bradford protein assay. The flea body extract was fractioned by ultracentrifugation using Amicon® tubes (Millipore, Billerica, MA, USA) on three fractions including different proteins, comprised between 0–50 kDa, 50–100 kDa and >100 kDda molecular weight ranges. For assays, it was used a flea extract concentration of 20 μg/mL based on a previous antigen titration curve.

### Basophil degranulation test

To establish if the IgE that recognizes flea antigens is functional and capable to induce basophil degranulation in patients and healthy controls, it was carry out a basophil degranulation test by flow cytometry using Basotest® kit (Orpegen Pharma, Heidelberg, Germany. This kit contains the chemotactic peptide N-formyl-Met-Leu-Phe (fMLP) used as a positive control, and a two-color antibody reagent (anti-IgE-PE/anti-CD63 FITC) for assessing the activation and degranulation of basophils. As a negative control was used the washing solution (from the kit). Test was done follow the directions from manufacturer.

### Characterization of cell populations by flow cytometry

To define the basophil population more precisely, we included for the surface staining, markers for molecules CD123-PerCP-Cy5,5 y HLA-DR-Pe-Cy7 (BD Biosciences, San José, CA, USA), in addition to the antibodies used to identify IgE and CD63 included in the Basotest® kit. The phenotype of the basophil population was defined as CD123^+^, IgE^+^, HLA-DR^-^ for this assay. Basophil degranulation was measured by over-expression of CD63, marker of late endosomes, which increases its expression in cell surface by fusion of the granules membranes with the plasmatic membrane. Cells were acquired in a flow cytometer FACSAria and analyzed with FACSDiva software (BD Biosciences). At least 8x10^5 ^cells were analyzed for each experiment.

### Statistical analysis

A descriptive analysis was carried out on cell populations using percentages, medians and standard deviations. Differences among groups were determined by U Mann–Whitney using GraphPad Prism 5.0. software. Differences were considered statistically significant when p < 0.05.

## Results

**Figure 1:**
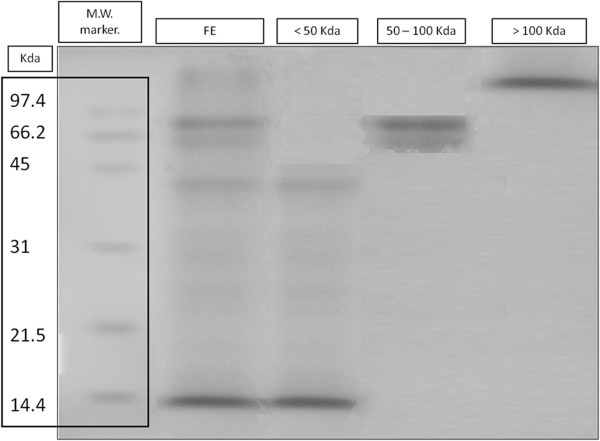
**Evaluation of efficiency of the fractionation of flea extract by SDS-PAGE.** Electrophoretic pattern showed by three protein fractions (<50 Kda, 50–100 Kda and >100 Kda) and flea extract (FE). Coomasie blue staining.

The protein concentration from the flea extract was 1.3 mg/mL, and fractions obtained by ultracentrifugation showed ranges of molecular weight between 0–50 kDa, 50–100 kDa and >100 kDa (Figure 1).

**Figure 2:**
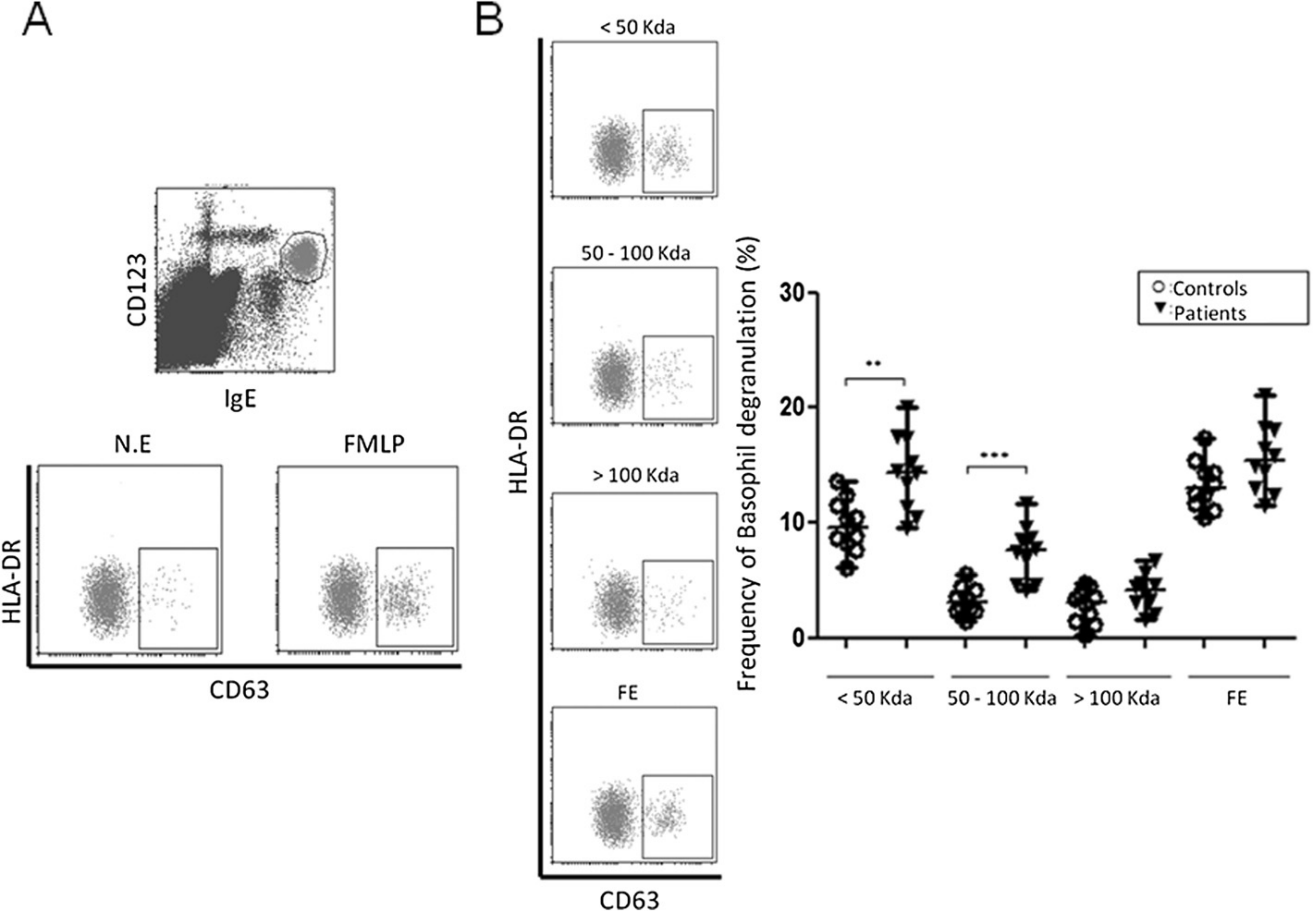
**Analysis of basophil degranulation induced by proteic fractions derived from flea extract.** Representative Dot Plot of the basophil degranulation induced by three protein fractions (<50 Kda, 50–100 Kda and >100 Kda) and flea extract (FE) (**A**). Frequency of basophil degranulation induced by protein fractions and FE in PUFB patients and healthy controls (**B**). ** p < 0.1, *** p < 0.005.

IgE reactivity to the fractions was tested *in vitro* by its capability to induce basophil degranulation. Basophil population was gated based on the expression of molecules CD123, IgE and HLA-DR. On this population, it was analyzed the over-expression of the marker CD63 as marker of degranulation (Figure 2A). Results showed that specific IgE from patients with PUFB and healthy controls induced a basophil degranulation response to the flea extract with no significant differences between them (16.2 ± 3.1% vs 13.6 ± 2.8% respectively p = 0.77) (Figure 2B).

However, significant differences were observed on the basophil degranulation response mediated by IgE, from patients compared with healthy controls to <50 kDa (14.9 ± 5.1% vs 9.7 ± 2.1% respectively p = 0.0058) and 50–100 kDa protein fractions (8.3 ± 3.2% vs 2.8 ± 1.6% respectively p = 0.0021) (Figure 2B).

## Discussion

After repeated exposure to a particular antigen, atopic people develop strong Th2 responses with IgE production. This specific IgE sensitizes mast cells and basophils by binding and up-regulation of their FcϵRI receptors. Subsequent exposures to the same antigen activate the mast cells and basophils resulting in pathological reactions of immediate hypersensitivity providing a mechanism for the amplification of IgE-mediated reactions [8].

*In vivo* properties from basophils have been ignored for a long time because their low frequency in peripheral blood and the absence of specific markers to identify them, but recently, it has been established the important role of these cells on the initial phases of the chronic allergic inflammation mediated by IgE in an independent way from T and mast cells [9,10] like orchestrators of the response to allergens by Th2 cells because their capacity to present antigens in the MHC class II molecules context [8,11,12] and cytokine production as response to IL-4 [8].

Histological analysis of infiltrates from skin pathologies have revealed presence of basophils in tissue lesions from patients with atopic dermatitis, prurigo, urticaria and insect bites but not in biopsies from *psoriasis vulgaris*, tumoral lesions, mastocytosis, systemic sclerosis and systemic lupus erythematosus [13]. Given the importance of basophils in chronic allergic inflammation and skin allergic diseases, it’s important to explore the role of this cell population in PUFB.

*In vitro* functional tests for diagnostic of allergic pathologies have been focused on basophils because, in contrast with mast cells, basophils are circulating in peripheral blood and they could be easier to isolate [14]. In this study was used a modified Basotest® protocol in which, in addition to the use of allergenic extracts from flea, made in our laboratory, were included the anti-CD123 and anti-HLA-DR fluorescent antibodies to define, more precisely, the basophil population present in peripheral blood.

CD123 is the low-affinity subunit of the IL-3 receptor and is expressed at high levels on plasmacytoid dendritic cells (pDC) and basophils. However, in contrast to basophils, pDC show a higher expression of HLA-DR molecules for their role in antigen presentation [15]. Although it has been proposed the use of other molecules as markers of activated basophils, selection based on CD63/CD123/HLA-DR expression provides an accurate method for basophil identification [16-18]. In our case, the combination of these markers allowed a better identification of the basophil population, that was the reason because we decided to modify the protocol and included antibodies directed to HLA-DR and CD123 molecules to characterize the basophil population. In addition, because our interest was the functional activity of IgE, we focused in the basophil population that carried IgE bind to their surface.

Allergen extracts are complex mixtures of macromolecules as proteins, glycoproteins and polysaccharides, this mixture contains the major, intermediate and minor allergens that must be identified to obtain clinically effective extracts for immunotherapy protocols and for allergy diagnosis [19,20].

In this study, our results show that the response derived from the interaction between allergens from flea extract and the IgE^+^ basophils was not different between PUFB patients and healthy controls. In contrast, when the protein fractions derived from the complete extract were used, we observed significant differences on the basophil degranulation response induced by proteins from 50–100 kDa and <50 kDa in the studied groups.

This results support the findings showing that the relevant allergens in the immuno-pathogenesis of the disease correspond to low molecular weight proteins, as previously was showed on the allergogram [4]. In addition, this is a first report of the functional capability from specific IgE antibodies to flea allergens to activate cells that are important in the allergic immune response such as basophils.

## Competing interests

The authors declare that they have no competing interests.

## Authors’ contributions

OD participated in the design of the study, carried out the flow cytometry assays, analysis and discussion of results and drafted the manuscript. SD participated in the flow cytometry assays, analysis and discussion of results and helped to draft the manuscript. EG participated in the design of the study, clinical diagnosis of patients, analysis and discussion of results and drafted the manuscript. EH participated in clinical diagnosis of patients and helped to draft the manuscript. AC participated in the design of the study, analysis and discussion of results and helped to draft the manuscript. AR participated in the design of the study, consecution and administration of financial resources, analysis and discussion of results and drafted the manuscript. All authors read and approved the final manuscript.
